# The Effect of Spanwise Folding on the Aerodynamic Performance of a Passively Deformed Flapping Wing

**DOI:** 10.3390/biomimetics9010042

**Published:** 2024-01-10

**Authors:** Ming Qi, Menglong Ding, Wenguo Zhu, Shu Li

**Affiliations:** 1 School of Aeronautic Science and Engineering, Beihang University, Beijing 100191, China; 2 Tianmushan Laboratory, Hangzhou 310023, China; bht0024@tmslab.com; 3 Norinco Group Institute of Navigation and Control Technology, Beijing 100191, China; zhuwenguo@buaa.edu.cn

**Keywords:** folding motion, flapping wing, fluid–structure coupling, unsteady panel method, propulsion efficiency

## Abstract

The wings of birds exhibit multi-degree-of-freedom motions during flight. Among them, the flapping folding motion and chordwise passive deformation of the wings are prominent features of large birds in flight, contributing to their exceptional flight capabilities. This article presents a method for the fast and accurate calculation of folding passive torsional flapping wings in the early design stage. The method utilizes the unsteady three-dimensional panel method to solve the aerodynamic force and the linear beam element model to analyze the fluid–structure coupling problem. Performance comparisons of folding flapping wings with different kinematics are conducted, and the effects of various kinematic parameters on folding flapping wings are analyzed. The results indicate that kinematic parameters significantly influence the lift coefficient, thrust coefficient, and propulsion efficiency. Selecting the appropriate kinematic and geometric parameters is crucial for enhancing the efficiency of the folding flapping wing.

## 1. Introduction

Unlike fixed-wing aircraft, flapping wings have numerous degrees of freedom, encompassing various motions and deformations [1], such as active flapping and twisting, passive torsional and bending deformations, and spanwise folding. These dynamic features enable flapping wings to efficiently generate lift and thrust, thereby enhancing flight performance [2]. Consequently, comprehending the impact of wing motions on flight performance is pivotal in flapping-wing design. Given that the spanwise folding motion is a primary characteristic of large birds in flight [3], this paper predominantly investigates the influence of the folding motion combined with the wing’s passive torsional deformation on performance.

Flapping wings with folding motions have been studied. Send [4] designed the famous smart bird and utilized thin-plate theory and CFD software to study the unsteady two-dimensional airfoil. Finally, experiments were applied to the analysis. Kim [5] built a folding bird-like flapping wing, analyzed its performance using simple methods, and conducted flight tests. Huang [6] employed the two-dimensional airfoil method to solve the folding flapping wing’s aerodynamic forces and optimized the structural parameters in the linkage mechanism. Han [7] applied the panel method to study the unsteady aerodynamic force of the flapping wing and found that, compared with simple up-and-down flapping, the wing combined with the folding motion can obtain greater lift and thrust, but the wing twist was not considered. Yang [8] employed the unsteady vortex lattice method to calculate the aerodynamic force of the folding ornithopter and employed an optimization algorithm to optimize the shape and motion parameters to maximize the average lift. Karimian [9] applied a simple two-dimensional lift line model and a linear beam element model to study the aeroelastic model of a flapping wing with folding. He found that a wing with folding motion can improve flight performance and propulsion efficiency, and that the phase of the movement of the outer wing has a great influence on aerodynamic performance. Verstraete [10] utilized the unsteady vortex lattice method and a linear finite element model to simulate a seagull-like flapping wing with folding and studied the effect of folding on the flutter speed. Chang [11] applied the method of solving the Navier–Stokes equations to calculate the folding-flapping-wing model. The torsional deformation in the model was preset without the fluid–structure interaction calculation. He found that lift and thrust were mainly generated in the downward-flapping stage. Lang [12] applied commercial software to solve N-S equations to study the effects of the folding parameters of a flat-panel wing on its performance. Bie [13] also applied a commercial N-S equation solver to study a bat-like folding flapping wing and analyzed the effects of the inner-to-outer wing ratio. Ryu [14] experimentally studied the folding flapping wing with a four-bar linkage mechanism and concluded that the folding motion of the wing is conducive to the generation of lift. Chen [15] employed an experimental method to study the influence of wing-folding amplitudes on the vertical aerodynamic force of a small flapping wing. Qin [16] employed a PIV experiment to study the evolution and physical characteristics of the tip vortex of a folding flapping wing.

The existing research exhibits numerous shortcomings. The design and manufacture of folding flapping wings have basically relied on experiments and simple analysis methods, which makes it difficult to guide the design. Regarding analytical approaches, the single-layer vortex lattice method is unable to compute the surface pressure coefficient; it can only assess changes in lift and thrust, leaving the wing surface state unclear. However, the surface pressure coefficient significantly influences the airfoil lift–drag ratio [17]. Although the method of solving the N-S equations can accurately determine the aerodynamic force, it is time-consuming and unsuitable for the initial design stage. Considering the fluid–structure coupling problem further exacerbates the computational time, rendering it impractical. Regarding research problems, the impact of folding on propulsion efficiency has not been considered. Propulsion efficiency is, in fact, crucial for achieving efficient flight. Studies seldom consider the effects of wing torsional deformation and the geometric parameters of the wing itself, concentrating solely on the impact of the folding motion. This oversight results in widespread separation on the wing surface, deviating from real-world scenarios [18].

**Figure 1:**
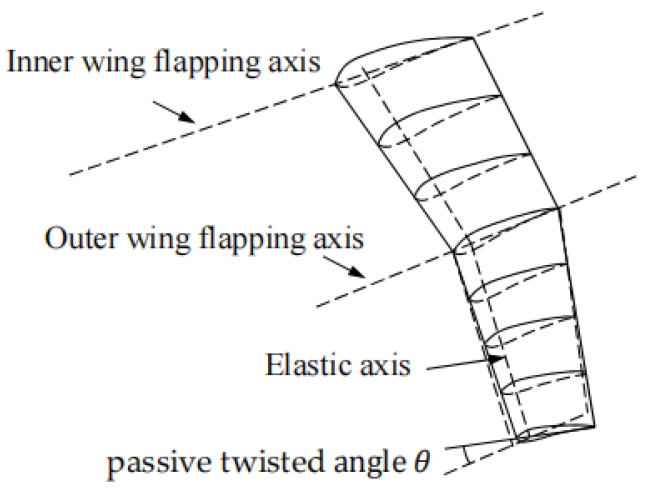
Passive torsional deformation of folding flapping wing.

To address the shortcomings of current research, this article proposes a method for solving the fluid–structure coupling problem of folding flapping wings. The unsteady three-dimensional panel method is employed to calculate the wing surface pressure coefficient and aerodynamic parameters, while the linear beam element model is utilized to account for the outer wing’s passive torsional deformation, as shown in Figure 1. A kinematic analysis of folding flapping wings with varying kinematic parameters is conducted. A dynamic analysis of folding flapping wings with different parameters undergoing passive twisting due to aerodynamic forces is performed. Performance parameters such as the lift coefficient, thrust coefficient, and propulsion efficiency are compared and analyzed. Various aerodynamics throughout the period are compared, and the wing surface pressure coefficients are analyzed. The influence of different parameters on the aerodynamic performance of folding flapping wings is discussed, their mechanism is analyzed, and design guidance is provided in the conclusion.

## 2. Theoretical Methods

### 2.1. Aerodynamic Model

This article employs the unsteady panel method to calculate the 3D aerodynamic force. The panel method is an inviscid method that has been studied by many researchers. Magnus [19] and Ashby [20] utilized high-order and low-order surface element methods to calculate 3D aerodynamic forces, respectively. Similarly, Maskew [21] and Johnson [22] utilized the panel method to establish a general program for solving wing aerodynamic forces. Vest [18] utilized the panel method to study the flight performance of birds. The method in this article originates from Hess [23], and Hess’s method is extended to unsteady situations.

**Figure 2:**
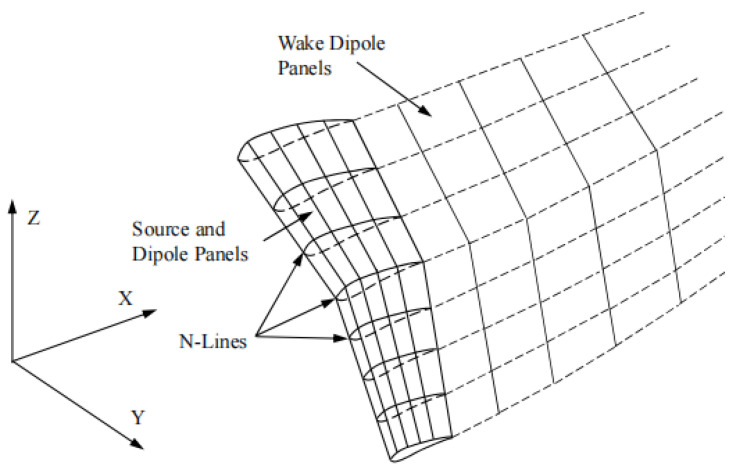
The 3D unsteady panel method.

As shown in Figure 2, the panel model consists of a wing panel and a wake panel. The wing panel consists of a source panel and a dipole panel, and the wake panel consists of only the dipole panel. The wing is divided into M portions in the chord direction and N portions by N-lines in the spanwise direction. To save calculation time, the free-wake model is not used, but the fixed-wake model described by Reichert [24] is employed. 

The velocity (Neumann) boundary condition is employed as the boundary condition, which means that the velocity normal to the wing surface is zero. For unsteady conditions, the trailing edge of the wing must satisfy the unsteady Kutta condition [25], which is

(1) $${\left[V_{up}^{t}\right]}_{k}^{2}-{\left[V_{down}^{t}\right]}_{k}^{2}=2{\left[\frac{\partial \left(\Phi_{down}-\Phi_{up}\right)}{\partial t}\right]}_{k}$$

where $V^{t}\;$ is the tangential velocity, and Φ is the velocity potential of the trailing edge at time step *k.* More details can be found in Reference [26].

### 2.2. Structural Model

To simulate the torsional deformation of the wing beam, a linear beam element model is utilized. The local element stiffness matrix is $\left[K\right]$:

$$\left[K\right]=\left[\begin{array}{cccccc} \frac{12EI}{l^{3}} & 0 & \frac{-6EI}{l^{2}} & \frac{-12EI}{l^{3}} & 0 & \frac{-6EI}{l^{2}}\\ 0 & \frac{GJ}{l} & 0 & 0 & \frac{-GJ}{l} & 0\\ \frac{-6EI}{l^{2}} & 0 & \frac{4EI}{l} & \frac{6EI}{l^{2}} & 0 & \frac{2EI}{l}\\ \frac{-12EI}{l^{3}} & 0 & \frac{6EI}{l^{2}} & \frac{12EI}{l^{3}} & 0 & \frac{6EI}{l^{2}}\\ 0 & \frac{-GJ}{l} & 0 & 0 & \frac{GJ}{l} & 0\\ \frac{-6EI}{l^{2}} & 0 & \frac{2EI}{l} & \frac{6EI}{l^{2}} & 0 & \frac{4EI}{l} \end{array}\right]$$

where *EI* is the bending stiffness and *GJ* is the torsional stiffness of an element, and *l* is the length of an element.

### 2.3. Aeroelastic Coupling

The unsteady aerodynamic force, the wing mass’s inertial force, and the beam’s deformation internal force all achieve a dynamic balance during the flapping of the wings. There is an interaction between the deformation of the wing and aerodynamic forces. Given the data at time step *k*, the equilibrium equation at time step *k* + 1 can be solved:

(2) $$\left[K\right]{\vec{q}}_{k+1}+\left[M\right]{\ddot{\vec{q}}}_{k+1}={\vec{F}}_{k+1}$$

where $\left[K\right]\;$ is the stiffness matrix, $\left[M\right]\;$ is the mass matrix, ${\vec{q}}_{k+1}$ is the deformation displacement, and ${\vec{F}}_{k+1}$ is the force and moment on the beam. The Newmark-*β* method [27] is utilized to solve this second-order equation. This method is unconditionally stable.

(3) $${\dot{\vec{q}}}_{k+1}={\dot{\vec{q}}}_{k}+\left[\left(1-\delta \right){\ddot{\vec{q}}}_{k}+\delta {\ddot{\vec{q}}}_{k+1}\right]∆t$$

(4) $${\vec{q}}_{k+1}={\vec{q}}_{k}+{\dot{\vec{q}}}_{k}∆t+\left[\left(0.5-\lambda \right){\ddot{\vec{q}}}_{k}+{\ddot{\beta \vec{q}}}_{k+1}\right]{∆t}^{2}$$

### 2.4. Performance Parameters

The unsteady Bernoulli theorem [28] states that the pressure coefficient in unsteady flows can be written as follows:

(5) $$C_{p}=\frac{p-p_{\infty }}{\left(1/2\right)\rho V_{\infty }^{2}}={\left(\frac{V_{move}}{V_{\infty }}\right)}^{2}-{\left(\frac{V}{V_{\infty }}\right)}^{2}-\frac{2}{V_{\infty }^{2}}\frac{\partial \Phi }{\partial t}$$

where *V* is the velocity of the wing, $V_{\infty }$ is the far-field velocity, Φ is the velocity potential, and $V_{move}$ is the motion velocity of the wing with respect to the global coordinate system. The outer wing performs a pitching motion around the outer wing beam, accompanied by a flapping motion around the axis of the tip of the inner wing, while the inner wing flaps around the axis of the inner wing root. The torsional moment $M_{t}\left(t\right)$, bending moment $M_{b}\left(t\right)$, vertical lift $F_{z}\left(t\right)$, and horizontal thrust $F_{x}\left(t\right)$ are all produced by the moving wing. The wing’s output of power is as follows:

(6) $$P\left(t\right)=M_{b\_in}\left(t\right)\cdot {\dot{\beta }}_{in}\left(t\right)+M_{b\_out}\left(t\right)\cdot {\dot{\beta }}_{out}\left(t\right)$$

As shown in Figure 1, the wing consists of two parts: the inner wing and the outer wing. $\beta_{in}\left(t\right)$ is the flapping angle of the inner wing, and $\beta_{out}\left(t\right)$ is the flapping angle of the outer wing. The bending moment at the wing root is $M_{b\_in}\left(t\right)$, while the bending moment at the outer wing root is $M_{b\_out}\left(t\right)$.

$\bar{F_{x}}$, $\bar{F_{z}}$, and $\bar{P}$ are the following time-averaged quantities:

(7) $$\bar{F_{x}}=\frac{1}{T}\int_{0}^{T}F_{x}\left(t\right)dt$$

(8) $$\bar{F_{z}}=\frac{1}{T}\int_{0}^{T}F_{z}\left(t\right)dt$$

(9) $$\bar{P}=\frac{1}{T}\int_{0}^{T}P\left(t\right)dt$$

The dimensionless forms of these quantities are

(10) $$C_{t}=\frac{\bar{F_{x}}}{1/2\rho V_{\infty }^{2}S}$$

(11) $$C_{l}=\frac{\bar{F_{z}}}{1/2\rho V_{\infty }^{2}S}$$

(12) $$C_{P}=\frac{\bar{P}}{1/2\rho V_{\infty }^{3}S}$$

Propulsion efficiency is defined as

(13) $$\eta_{P}=\frac{C_{t}}{C_{P}}$$

### 2.5. Solver Validation

#### 2.5.1. Aerodynamic Force

**Figure 3:**
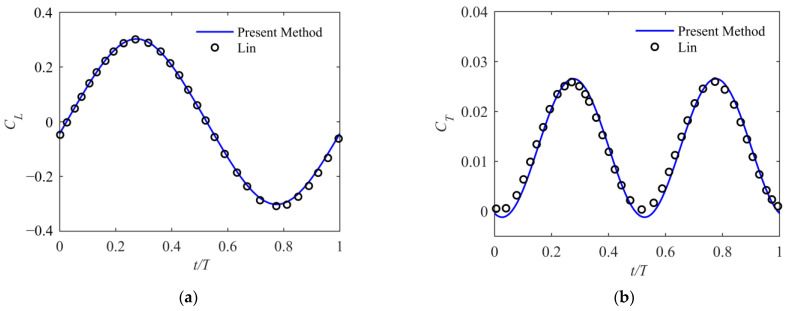
Comparison of calculation results with Lin’s calculations. (**a**) Lift coefficient; (**b**) thrust coefficient.

The unsteady aerodynamic force obtained with the present method was validated with the numeral calculation described by Lin [29] on a flapping rectangular wing with the airfoil NACA0014. It is solved by using the 3-dimensional Euler equations. The wing’s maximum twisting angle is 4 degrees at the wing tip, and active twisting varies linearly along the span with a phase lag of 90 degrees. As shown in Figure 3, the method employed in this article yields results that closely match Lin’s calculations.

#### 2.5.2. Fluid–Structure Coupling

The aeroelastic response obtained with the present method was validated with the flapping wing studied by DeLaurier [30]. DeLaurier performed computations and experiments with the flapping wing he designed. Similar to the flapping wing in this article, DeLaurier’s flapping wings are also passively twisted. All parameters are provided in Reference [30] to facilitate the comparison of methods.

**Figure 4:**
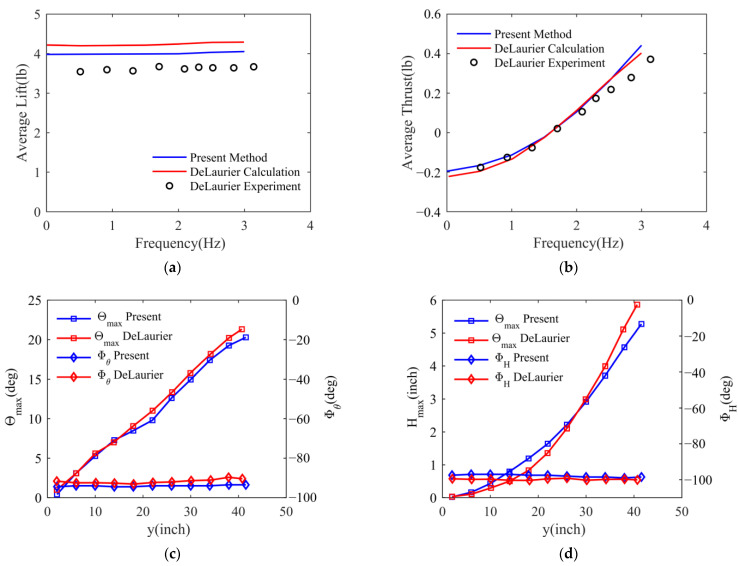
Comparison of calculation results with Delaurier’s calculation and experimental results. (**a**) Average lift; (**b**) average thrust; (**c**) torsion amplitude and phase angle; (**d**) flapping elastic amplitude and phase angle.

As shown in Figure 4a,b, compared with the calculations used by DeLaurier, the lift coefficient and thrust coefficient calculated using this method are in good agreement with the experiment. Since the non-stick model is used, there are still some differences, especially when trailing-edge separation occurs on the outer wing after the period becomes small.

As shown in Figure 4c, the maximum twist angle amplitudes $\Theta_{max}$ along the inner wing are almost the same, but there are some differences in the outer wing sections. The phase angles $\Phi_{\theta }$ between the twisting angle and flapping motion are slightly larger than the results of DeLaurier. As shown in Figure 4d, compared to DeLaurier’s results, the flapping elastic amplitudes $H_{max}$ are larger in the inner wing but smaller in the outer wing, and their phase angles $\Phi_{H}$ are marginally smaller. These deviations could result from a mix of variations in the structural and aerodynamic models. 

## 3. Wing Model

### 3.1. Wing Calculation Model

The wings of the folding flapping wing are divided into two sections, namely, the inner wing and the outer wing. The inner section is torsionally rigid and does not twist with flapping, while the outer wing passively twists with flapping. At the same time, the outer wing section performs a flapping motion relative to the inner section.

**Figure 5:**
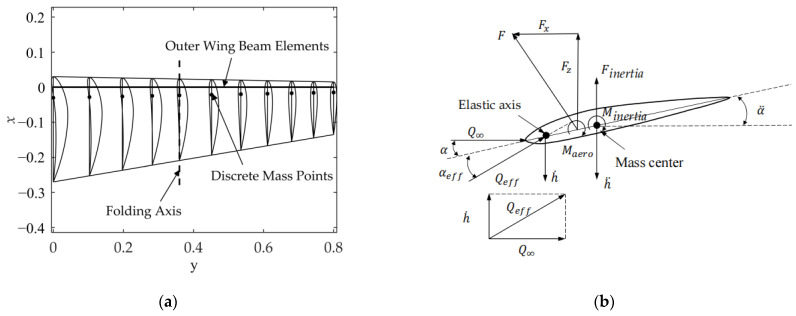
Wing model. (**a**) Structure of the wing; (**b**) forces and moments on the airfoil.

In this article, inertia force and aerodynamic force are responsible for the wing’s passive torsion. The lift center on the airfoil is around 25% of the chord length to utilize the aerodynamic force to produce torque on the beam as much as possible, and considering the issues of the wing structure, the beam is placed at 10% of the chord line. It is assumed that the beam is the only source of torsional stiffness. As shown in Figure 5a, the beam elements are distributed into N portions along the span direction, which is consistent with the aerodynamic panel. It is assumed that the wing’s mass is distributed by volume and is discretely separated into mass locations at 10% of the chord behind the beam on the chord line. As shown in Figure 5b, the forces and moments in the 2D wing profile, which conducts pitching and plunging motions along the elastic axis, are depicted. The effective angle of attack equals the angle of incidence plus the self-motion.

### 3.2. Structural and Kinematic Parameters

**Figure 6:**
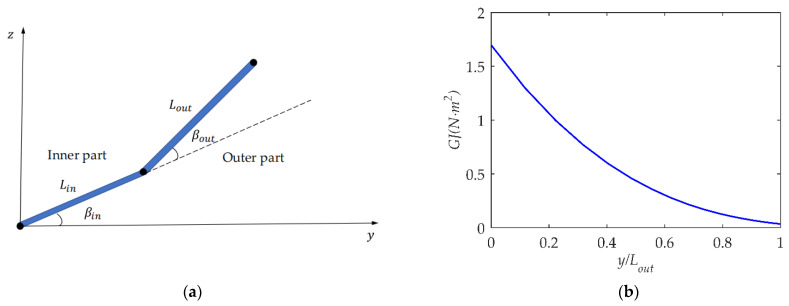
Structural and kinematic parameters of folding flapping wing. (**a**) Flapping angle of folding wing; (**b**) torsional stiffness of outer wing.

The shape of the wing is inspired by the Canadian goose, which has a half wingspan of 0.8 m, an aspect ratio of 12, a root chord length of 0.3 m, and a root-to-tip ratio of 2. The flight velocity is 15 m/s, and the Reynolds number of the wing is from 367,500 to 183,750. The flapping period is 0.33 s, the wing-tip Strouhal number *St* = 0.167, and the wing-tip reduced frequency *k* = 0.0952 can be calculated. As shown in Figure 5a, the wing-root and wing-tip airfoils are NACA8412 and NACA0012, respectively, and the remaining portion of the wing has a linearly interpolated transition from the wing root to the wing tip according to the parameters of the NACA airfoil family. The root airfoil’s angle of incidence is 4 degrees. The airfoil must function within an appropriate angle of attack if the flapping wing is intended to generate enough lift and thrust and have good propulsion efficiency [18,31]. To balance the manufacturing difficulty and ensure that the wing has a suitable torsion law, it is assumed that there is a linear relationship between the beam’s cross-sectional diameter and wingspan; thus, the torsional stiffness is supposed to vary with the wingspan to the fourth power. As shown in Figure 6b, the torsional stiffness GJ represents torsional stiffnesses along the outer wing. Since this article discusses the effect of spanwise folding on a flapping wing, the bending stiffness is assumed to be rigid to exclude interference.

The lengths of the inner and outer sections are, respectively, 0.45 and 0.55 of the total span. For the convenience of discussion, the flapping motion of the inner/outer wing is a simple harmonic motion. As shown in Figure 6a, the flapping motion law of the inner wing is

(14) $$\beta_{in}\left(t\right)=\beta_{in\_max}cos\left(\omega t\right)$$

and the flapping motion law of the outer wing relative to the inner wing is

$$\beta_{out}\left(t\right)=\beta_{mean}-\beta_{out\_max}cos\left(\omega t-\phi_{\beta }\right)$$

where $\beta_{in\_max}$ is the flapping amplitude of the inner wing, $\beta_{mean}$ is the mean folding angle, $\beta_{out\_max}$ is the folding angle amplitude of the outer wing, and $\phi_{\beta }$ is the phase difference between the flapping angles of the inner and outer wings. In order to show the characteristics of folding flapping wings, $\beta_{out\_max}=30{}^{\circ}$ is used, and the other parameters are studied with different values.

## 4. Results and Discussion

To study the impact of different parameters on flight performance, a kinematic analysis of folding flapping wings with different kinematic parameters was conducted based on real bird flight postures. The flapping phase angles $\phi_{\beta }$ are 0, 45, and 90 degrees, the inner wing flapping angles $\beta_{in\_max}$ are 20 and 30 degrees, and the average folding angles $\beta_{mean}$ are 0 and 30 degrees. The different combinations are Case 1 to Case 9, as shown in Table 1.

### 4.1. Kinematic Analysis

#### 4.1.1. Flapping Trajectory of the Folding Flapping Wing

**Figure 7:**
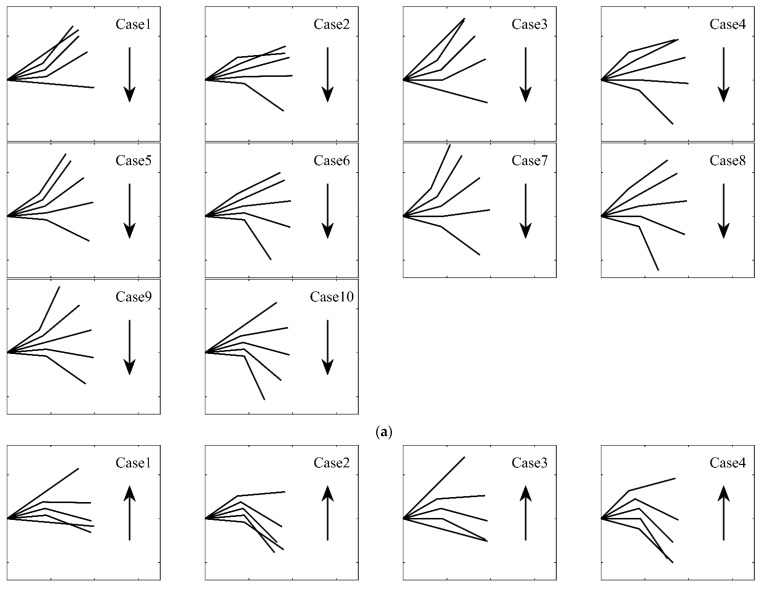

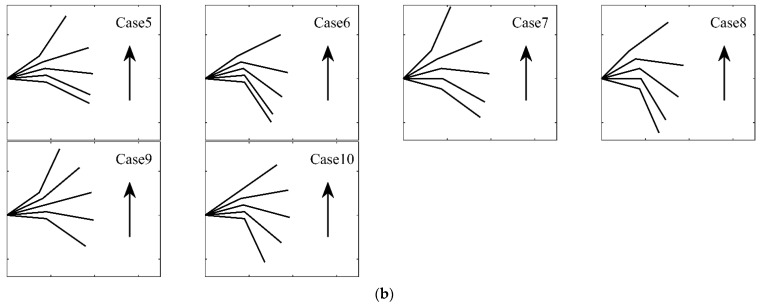
The trajectory of folding wing during the period. (**a**) The trajectory of downward flapping from Case 1 to Case 10; (**b**) the trajectory of upward flapping from Case 1 to Case 10.

The trajectories of the folding flapping wing during flapping are different due to different kinematic parameters. Figure 7a shows the trajectory of the downward-flapping stage in Cases 1 to 10, and Figure 7b shows the trajectory of the upward-flapping stage in Cases 1 to 10.

It can be seen that the average folding angle $\beta_{mean}$ affects the configuration of the folded wing during flapping. When $\beta_{mean}=0{}^{\circ}$, the configuration of the wing at the highest and lowest points is symmetrical with respect to half of the inner wing flapping angle, as in Cases 1, 3, and 5. It is asymmetric when $\beta_{mean}$ is 3$0^{{}^{\circ}}$, as in Cases 2, 4, and 6. We expect that the trajectory will be different during upward and downward flapping. If the outer wing folds downward during upward flapping, the upward speed of the wing can be reduced, and the loss of lift during upward flapping can be reduced. The configurations of real large birds are asymmetric during flight. 

As shown in Cases 1, 5, and 9, when the flapping angular phase $\phi_{\beta }$ is small, the range of movement and the unsteadiness of the outer wing become large. And the smaller $\phi_{\beta }$ is, the more similar the trajectories of the wing are during upward and downward flapping. For example, the upward and downward trajectories in Cases 9 and 10 are exactly the same, while the upward and downward trajectories in Cases 1 and 2 are different. It can be seen from the density of the trajectory that the Case 1 and 2 velocities will be greater in the rear part of the downward and upward flapping than that in the front part.

When the maximum flapping angle $\beta_{in\_max}$ is large, the overall flapping angle is large, and the unsteadiness of the entire wing is enhanced.

#### 4.1.2. Velocity of Wing Tip

**Figure 8:**
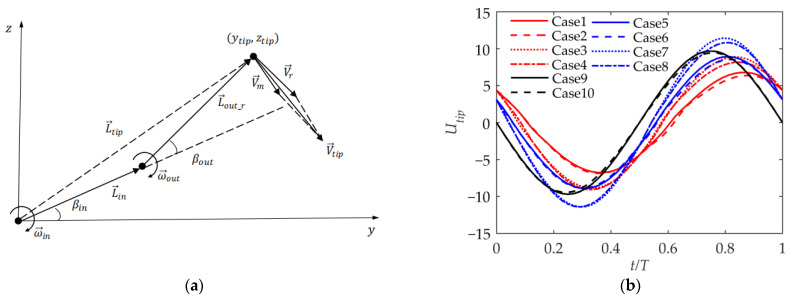
Velocity of wing tip. (**a**) The synthesis of wing-tip flapping velocity; (**b**) wing-tip flapping velocity.

The wing is composed of two sections, and the outer section of the wing rotates around the end of the inner section of the wing. As shown in Figure 8a, the flapping velocity at the wing tip is the sum of the migration velocity ${\vec{V}}_{m}$ and the relative velocity ${\vec{V}}_{r}$. The coordinate of the wing tip at time *t* is ${\vec{L}}_{tip}={(y}_{tip},z_{tip})$, and the migration velocity of the wing tip linked to the local coordinate system of the inner wing in the current wing configuration is as follows:

(15) $${\vec{V}}_{m}={\vec{\omega }}_{in}\times {\vec{L}}_{tip}$$

The relative velocity of the outer wing relative to the inner wing is

(16) $${\vec{V}}_{r}={\vec{\omega }}_{out}\times {\vec{L}}_{out\_r}$$

where

(17) $${\vec{L}}_{out\_r}={\vec{L}}_{tip}-{\vec{L}}_{in}$$

The sum of ${\vec{V}}_{m}$ and ${\vec{V}}_{m}$ is ${\vec{V}}_{tip}$:

(18) $${\vec{V}}_{tip}={\vec{V}}_{m}+{\vec{V}}_{r}$$

Figure 8b shows the flapping velocity of the wing tip with different kinematic parameters, where the positive and negative are based on the z-direction velocity. In general, the curve of the velocity change with time is similar to a sinusoidal curve. It can be seen that the larger the phase angle $\phi_{\beta }$, the smaller the velocity peak, which is similar to the trajectory analysis. And the larger the $\phi_{\beta }$, the more lagging the phase. The smaller $\phi_{\beta }$ is, the closer it is to a simple synthesis of the inner and outer wing velocities. According to the comparison between Cases 1 and 2, when $\beta_{mean}=30{}^{\circ}$, the velocity peak value is slightly less than or equal to that at $\beta_{mean}=0{}^{\circ}$, but the difference is tiny. Obviously, when $\beta_{n\_max}$ is larger, the wing-tip velocity is larger. In general, the phase angle $\phi_{\beta }$ has the greatest impact on the wing-tip velocity. 

### 4.2. Aerodynamic Performance 

For Case 1 to Case 10, the changes in the lift coefficient, thrust coefficient, and propulsion efficiency with the initial geometric twist angle of the wing were studied. The initial geometric twist angle is the twist change in the airfoil’s angle of incidence from the wing tip to the wing root during initial manufacturing, and the initial geometric twist angle varies linearly along the wingspan. 

**Figure 9:**
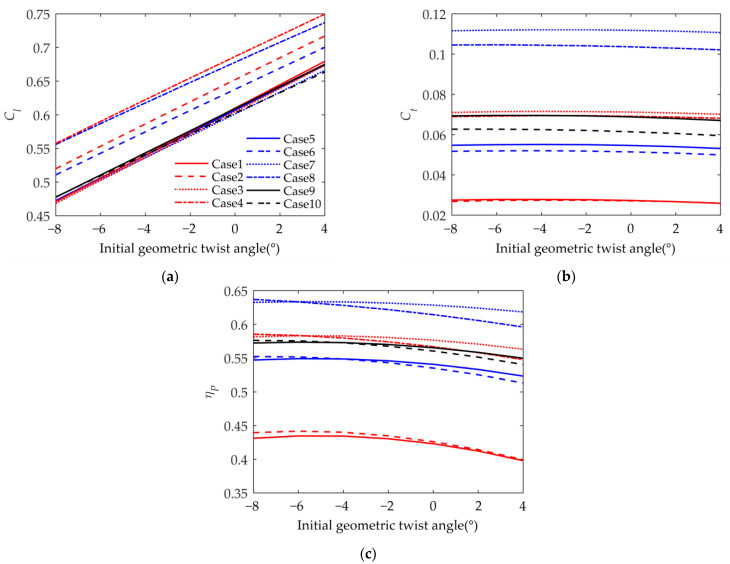
Variation in performance parameters with initial geometric twist angle. (**a**) Lift coefficient; (**b**) thrust coefficient; (**c**) propulsion efficiency.

As shown in Figure 9a, the lift coefficient increases as the initial geometric twist angle increases; this occurs because when the geometric twist angle increases, the average angle of attack of the airfoil increases. In Cases 1, 3, 5, 7, and 9, it can be seen that when $\beta_{mean}=0{}^{\circ}$, except for the initial geometric twist angle, the parameters have little impact on the lift coefficient. This occurs because when $\beta_{mean}=0{}^{\circ}$, the configuration of the two-section wing in the upward and downward phases is symmetrical. On the contrary, it can be seen in Cases 2, 4, 6, and 8 that the asymmetry of the wing configuration during the flapping period has a greater impact on the lift coefficient. The lift coefficient of the asymmetric configuration is larger, which is consistent with the analysis in Figure 7. Due to the difference in the inner wing flapping angle $\beta_{in\_max}$, the flapping trajectories during the cycle are different due to the asymmetry caused by $\beta_{mean}$. When $\beta_{in\_max}$ is larger, the lift coefficient will be larger, as shown in Cases 2 and 4. Phase $\phi_{\beta }$ has no obvious effect on the lift coefficient.

As shown in Figure 9b, the thrust coefficient first increases and then decreases as the initial geometric twist angle increases. This occurs because the airfoil has the strongest thrust generation capability near an average angle of attack of 0 degrees. The thrust coefficients are obviously divided into three levels, with the highest in Cases 7 and 8. The thrust coefficients in Cases 5 and 6 are slightly smaller than those in Cases 3, 4, 9, and 10, and those in Cases 1 and 2 are the smallest. It can be seen that $\beta_{mean}$ has a small impact on the thrust coefficient; the flapping angle $\beta_{in\_max}$ and phase $\phi_{\beta }$ have a greater impact on the thrust coefficient. This is consistent with the discussion of the wing-tip flapping velocity. The thrust coefficients in Cases 3 and 4 are larger than those in Cases 1 and 2 with the same $\phi_{\beta }$, which means that the thrust coefficient is proportional to the $\beta_{in\_max}$ angle. This occurs because a large flapping angle indicates a high flapping velocity and strong unsteadiness. With the same $\beta_{in\_max}$, the thrust coefficients in Cases 5 and 6 are larger than those in Cases 1 and 2. Similar to the wing-tip flapping velocity analysis, a smaller phase angle $\phi_{\beta }$ of the outer wing means a higher flapping velocity. The thrust coefficient is generally proportional to the flapping velocity of the outer wing.

As shown in Figure 9c, the propulsion efficiency first increases and then decreases as the initial geometric twist angle increases. The propulsion efficiency distribution is similar to the distribution of the thrust coefficient. The propulsion efficiency is high when the thrust coefficient is big. A high thrust coefficient means a large maximum angle of attack of the airfoil during the period. At high angles of attack, the lift-to-drag ratio of the airfoil will drop sharply because of the airflow separation. The aerodynamic calculation method in this article is an inviscid method that cannot simulate separation, but with the help of the airfoil surface pressure, this can be discussed.

### 4.3. Parameters within a Cycle

In order to further study the influence of kinematic parameters on folding flapping wings, the changes in aerodynamic parameters during the period were examined. The initial geometric twist angle of the wing was set to −4 degrees.

**Figure 10:**
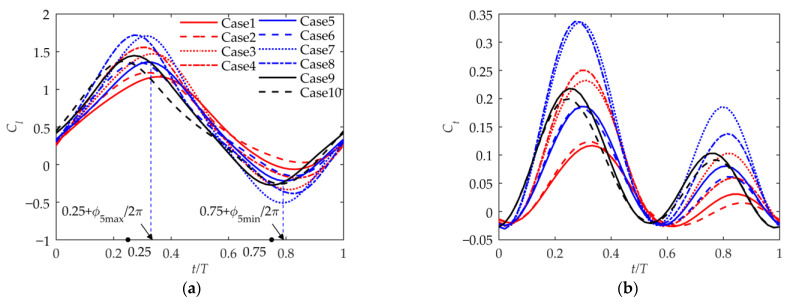
Lift and thrust coefficients during the period. (**a**) Lift coefficient; (**b**) thrust coefficient.

As shown in Figure 10a, the lift coefficient curve during the entire period is similar to a sinusoidal curve. The lift coefficient is generally positive, but it is negative for a short period during the upward phase. According to Cases 1, 5, and 9 or Cases 2, 6, and 10, the larger the phase angle $\phi_{\beta }$, the more lagging the phase of the lift coefficient, and the smaller the peak value of the lift coefficient. This is consistent with the wing-tip velocity shown in Figure 8b. Comparing Cases 1 and 2, Cases 5 and 6, or Cases 9 and 10, it can be seen that when $\beta_{mean}=30{}^{\circ}$, the phase of the maximum lift coefficient is advanced over that when $\beta_{mean}=0{}^{\circ}$, but the phase of the minimum lift coefficient lags behind that when $\beta_{mean}=0{}^{\circ}$. And it can be seen that the lift coefficient is larger when $\beta_{mean}=30{}^{\circ}$, which is consistent with the conclusion in Figure 10a. The $\beta_{mean}$ significantly affects the lift coefficient during the period. During the upward-flapping phase, the lift loss is reduced due to the wing’s folding motion. It can be seen from Cases 1 and 3 or Cases 5 and 7 that a larger $\beta_{in\_max}$ means a larger range of the lift coefficient curve, and $\beta_{in\_max}$ has little impact on the change in phase.

As shown in Figure 10b, the thrust coefficient curves have two peaks during the period. The peak value in the downward phase is greater than the peak value in the upward phase. The thrust coefficient is generally positive. Comparing Cases 1, 5, and 9 or Cases 2, 6, and 10, it can be seen that, similar to the case of the lift coefficient, the smaller the flapping phase angle $\phi_{\beta }$, the larger the thrust coefficient, and the more lagging the phase of the thrust coefficient. Comparing Cases 1 and 2, Cases 5 and 6, or Cases 9 and 10, it can be seen that when $\beta_{mean}=30{}^{\circ}$ and $\phi_{\beta }\neq 0{}^{\circ}$, the thrust coefficient is slightly larger than that when $\beta_{mean}=0{}^{\circ}$ in the downward phase but is smaller than that when $\beta_{mean}=0{}^{\circ}$ in the upward phase. This shows that the asymmetric motion trajectory reduces the thrust during the upward-flapping phase and concentrates the generation of thrust in the downward-flapping phase. It can be seen from Cases 1 and 3 or Cases 5 and 7 that a larger $\beta_{in\_max}$ means a larger range of the thrust coefficient curve due to the increased overall unsteadiness. And $\beta_{in\_max}$ has little impact on the thrust coefficient curve phase.

**Figure 11:**
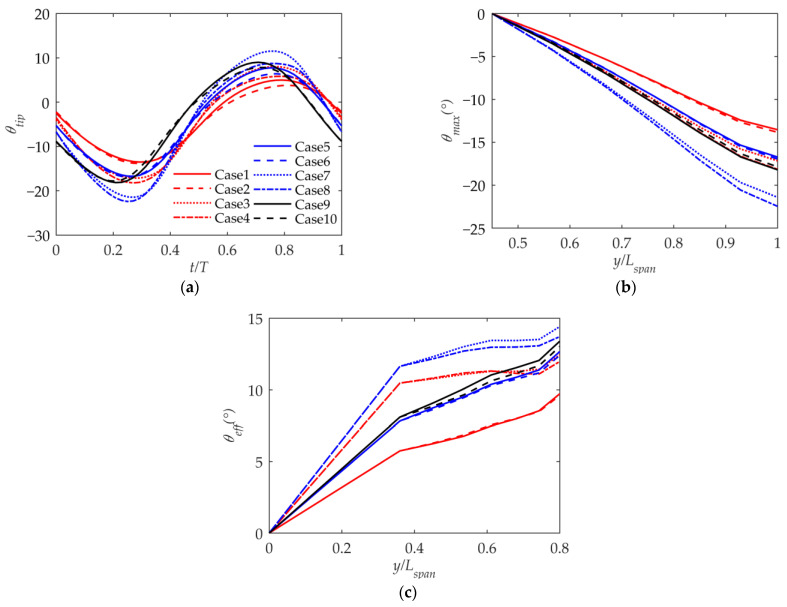
Twist angle and effective angle of attack along the span. (**a**) Wing-tip twist angle during the period; (**b**) maximum twist angles along the wingspan; (**c**) maximum effective angle of attack along the wingspan.

As shown in Figure 11a, there is some relationship between the wing-tip twist angle and lift coefficient curves. For different cases, when the range of the lift coefficient is large, the range of the negative wing-tip twist angle will also be large. And the phase of the wing-tip twist angle is consistent with the lift coefficient. The twist angle is positive during the upward phase of flapping and negative during the downward phase for the wing’s lift center is located behind the torsion center. The negative twist angles are significantly bigger than the positive twist angles throughout the period because of the angle of incidence and camber of the wing, which causes the positive lift to be substantially greater than the negative lift. The outer wing’s maximum twist angles are displayed along its wingspan in Figure 11b, where it can be seen that the twist angles are almost linear. Figure 11c shows the maximum effective angle of attack along the wingspan. It can be seen that the effective angle of attack of the inner wing increases linearly along the span, and due to the torsional deformation of the outer wing, there is a sudden change in the effective angle of attack of the outer wing and the inner wing. As shown in Figure 8b, there exists a correlation between the maximum equivalent angle of attack and the wing-tip flapping velocity, where a higher wing-tip flapping velocity corresponds to a higher effective angle of attack. The maximum effective angle of attack does not exceed 15 degrees, which shows that the method in this article is effective.

### 4.4. Pressure Coefficient 

In order to further study the influence of kinematic parameters on flight performance, the surface pressure coefficient of the airfoil at 75% of the span during the period was studied. Since the inner section of the wing does not twist, the pressure coefficient of the inner wing is not discussed. In order to further show the characteristics of the airfoil surface pressure coefficient, the pressure coefficient is studied at the maximum and minimum points of the lift coefficient curves, that is, $t/T=0.25+\phi_{max}/2\pi$ and $t/T=0.75+\phi_{min}/2\pi$. Due to the unsteadiness and the asymmetry of the folded-wing motion, the maximum and minimum lift coefficient points have phase angle deviations relative to the inner wing flapping angle, which are $\phi_{max}$ and $\phi_{min}$, respectively, as shown in Figure 10a. The other two moments in the cycle are set to $t/T=0.5+\left(\phi_{max}+\phi_{min}\right)/2\pi$ and $t/T=1+\left(\phi_{max}+\phi_{min}\right)/2\pi$.

**Figure 12:**
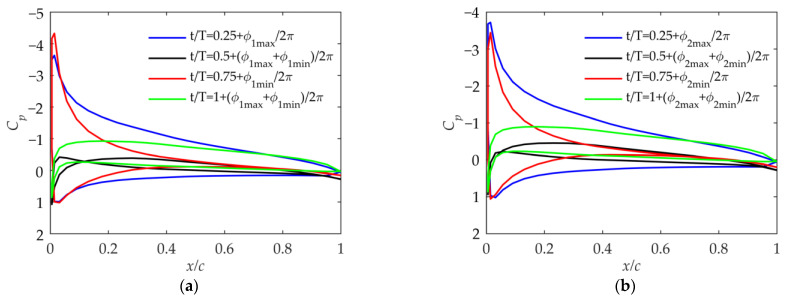
Pressure coefficient at 75% of the wingspan in Case 1 and Case 2. (**a**) Pressure coefficient in Case 1; (**b**) pressure coefficient in Case 2.

As shown in Figure 12, based on kinematic laws and the pressure coefficient distribution, the suction peak of the pressure coefficient distribution means that the positive effective angle of attack of the airfoil is large when $t/T=0.25+\phi_{max}/2\pi$ in Cases 1 and 2, and the negative effective angle of attack is large when $t/T=0.75+\phi_{min}/2\pi$. At the other two moments, the effective angle of attack of the airfoil is very small, generating almost no lift and thrust. Comparing Figure 12a,b, it can be seen that due to the different $\beta_{mean}$, the effective angle of attack of the airfoil in the cycle is greatly affected. Combined with Figure 8b and Figure 9a, it shows that although $\beta_{mean}$ has a small impact on the velocity, it can affect the effective angle of attack of the outer wing. Moreover, the average angle of attack during the period when $\beta_{mean}=30{}^{\circ}$ in Case 2 is biased toward the positive angle of attack compared to that when $\beta_{mean}=0{}^{\circ}$ in Case 1, which makes the lift coefficient larger.

**Figure 13:**
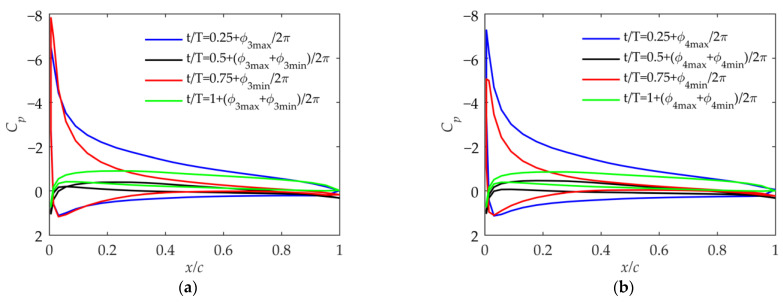
Pressure coefficient at 75% of the wingspan in Case 3 and Case 4. (**a**) Pressure coefficient in Case 3; (**b**) pressure coefficient in Case 4.

As shown in Figure 13, the effective positive angle of attack at $t/T=0.25+\phi_{max}/2\pi$ and the negative angle of attack at $t/T=0.75+\phi_{min}/2\pi$ in Cases 3 and 4 both increase significantly compared with those in Cases 1 and 2. The problem in Cases 3 and 4 is that there is always a moment when the effective angle of attack is excessively large during the upward or downward phase. This will aggravate the separation of the trailing edge of the airfoil, and the lift-to-drag ratio will drop sharply, thus reducing the propulsion efficiency. This situation should be avoided by adjusting the initial geometric twist angle.

**Figure 14:**
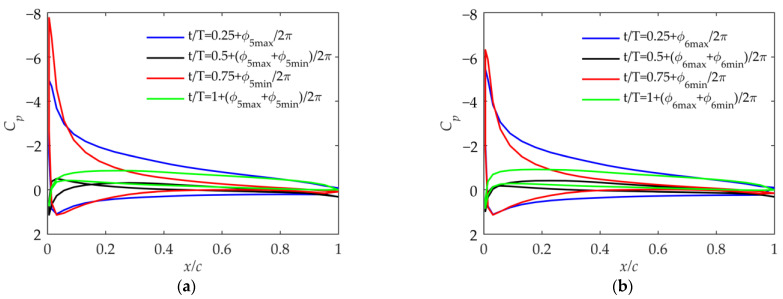
Pressure coefficient at 75% of the wingspan in Case 5 and Case 6. (**a**) Pressure coefficient in Case 5; (**b**) pressure coefficient in Case 6.

As shown in Figure 14, the airfoil surface pressure coefficient in Cases 5 and 6 represents that the effective-angle-of-attack range of the airfoil is larger than that in Cases 1 and 2. Due to the smaller flapping phase angle $\phi_{\beta }$, the flapping velocity of the outer wing is larger than that in Cases 1 and 2, which is consistent with the analysis in Figure 8b. And it can also be seen in Figure 9 that the thrust coefficient and propulsion efficiency are much larger than those in Cases 1 and 2. Comparing Cases 5 and 6 with Cases 1 and 2, the impact of $\beta_{mean}$ is the same.

**Figure 15:**
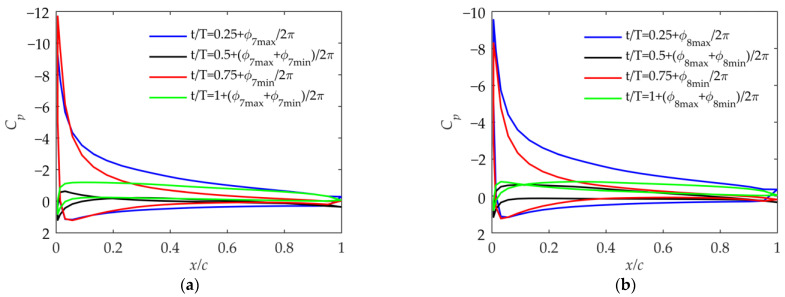
Pressure coefficient at 75% of the wingspan in Case 7 and Case 8. (**a**) Pressure coefficient in Case 7; (**b**) pressure coefficient in Case 8.

As shown in Figure 15, as $\beta_{in\_max}$ becomes larger, the pressure coefficient shows that the maximum effective angle of attack in Cases 7 and 8 becomes excessively large compared to that in Cases 5 and 6. From a practical perspective, such a large effective angle of attack of the outer wing is not allowed during the flapping process [18].

**Figure 16:**
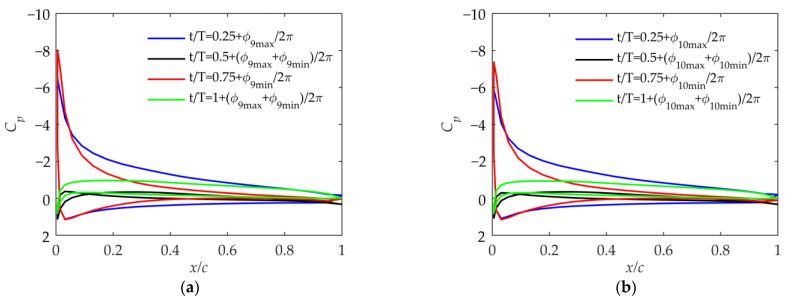
Pressure coefficient at 75% of the wingspan in Case 9 and Case 10. (**a**) Pressure coefficient in Case 9; (**b**) pressure coefficient in Case 10.

As shown in Figure 16, in Cases 9 and 10, the range of the effective angle of attack is large when the phase angle $\phi_{\beta }=0{}^{\circ}$ because the flapping velocity is large, according to Figure 8b. But according to Figure 9, its thrust and propulsion efficiency are smaller than those in Cases 3 and 4. Therefore, after paying the price of a large airfoil angle of attack and severe airflow separation, Cases 9 and 10 have no advantages over Cases 3 and 4 in the lift coefficient and thrust coefficient. When the flapping phase angle $\phi_{\beta }=0{}^{\circ}$, although it is beneficial to improve the flapping velocity of the outer wing, the outer wing is prone to have an excessive angle of attack, and the thrust coefficient and propulsion efficiency are not guaranteed.

**Figure 17:**
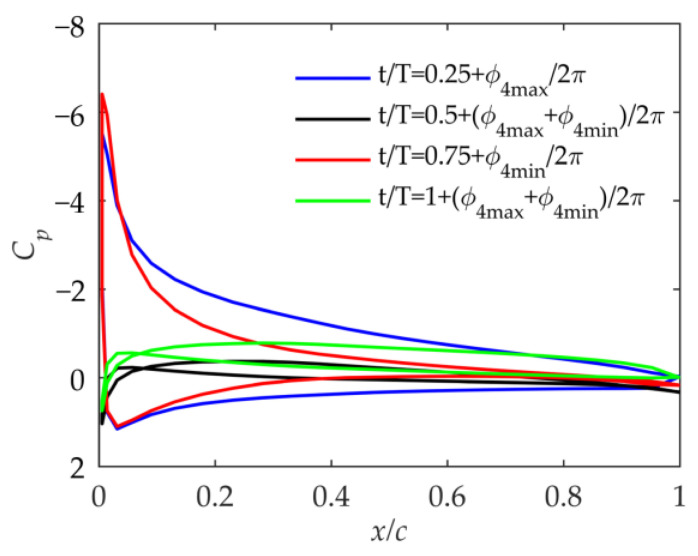
Pressure coefficient at 75% of the wingspan in Case 4 with initial geometric twist angle of −7 degrees.

According to the above discussion, Cases 3 and 4 have advantages over Cases 9 and 10. Now, compare Cases 3 and 4 with Cases 5 and 6. When the initial geometric twist angle is −4 degrees, there are some differences between the pressure coefficients in Figure 13 and Figure 14. In order to make the comparison more convincing, the geometric twist angle in Case 4 is set to −7 degrees. As shown in Figure 14b and Figure 17, the range of the airfoil pressure coefficient is almost the same, which means that the maximum effective angle of attack in the cycle is almost the same. According to Figure 9, it can be seen that the lift coefficient, thrust coefficient, and propulsion efficiency in Case 4 when the initial geometric twist angle is −7 degrees are greater than those in Case 6 when the initial geometric twist angle is −4 degrees. It can be concluded that a larger flapping phase angle $\phi_{\beta }$ has benefits, which can reduce the lift loss in the upward-flapping process of the folding flapping wing. 

The above-discussed results can be compared in Table 2 It can be seen that, excluding Cases 7 and 8, whose equivalent angle of attack is excessively large, Case 4, with a geometric twist angle of −7 degrees, has advantages over the other cases in terms of the lift coefficient, thrust coefficient, and propulsion efficiency. The same conclusion can also be learned from Figure 9.

## 5. Conclusions

This article proposes a method for the rapid and accurate calculation of folding passive torsional flapping wings during the early design stage. The unsteady three-dimensional surface element method is employed to compute aerodynamic forces, the linear beam element method is utilized to calculate torsional deformation, and the fluid–structure coupling calculation is conducted using the Newmark-β method. Using various kinematic parameters, this article investigates the changing rules of the lift coefficient, thrust coefficient, and propulsion efficiency as the initial geometric twist angle changes. It analyzes the lift coefficient, thrust coefficient, and torsional deformation of the wing throughout the entire period, along with the wing surface pressure coefficient at different moments. This article draws the following conclusions:

1. Kinematic parameters significantly impact performance. The flapping phase angle $\phi_{\beta }$ between the inner and outer wings affects the movement velocity of the outer wing. A larger $\phi_{\beta }$ implies a smaller flapping velocity for the outer wing, resulting in a smaller thrust coefficient and a more pronounced lag in the lift coefficient phase. A Larger $\phi_{\beta }$ also indicates asymmetry in the flapping trajectory.
2. The average folding angle $\beta_{mean}$ significantly influences the lift coefficient as it impacts the trajectory of the flapping wing. When $\beta_{mean}$ is positive, the folding motion is noticeable during the upward-flapping process, resulting in small lift loss and, consequently, a larger lift coefficient during this phase. It has a minor impact on the thrust coefficient and propulsion efficiency but greatly affects the effective angle of attack of the outer wing. $\beta_{mean}$ should be designed to align with the initial geometric twist angle. Otherwise, the airfoil’s angle of attack throughout the period might become excessive, leading to severe airflow separation and reduced propulsion efficiency.
3. The flapping angle $\beta_{in\_max}$ of the inner wing primarily influences the overall wing’s unsteadiness. An increase in $\beta_{in\_max}$ results in an elevation in the thrust coefficient, but it may lead to an excessive effective angle of attack for the outer wing, reducing the propulsion efficiency.
4. For folding flapping wings, there are principles for selecting kinematic parameters. From the perspective of the lift coefficient, the folding motion should be applied to reduce lift loss during the upward-flapping phase. The configuration of the wing during the period should be asymmetrical, and a large flapping phase angle $\phi_{\beta }$ and a positive average folding angle $\beta_{mean}$ should be selected. Regarding the thrust coefficient and propulsion efficiency, the generation of thrust should be concentrated in the downward-flapping phase when the lift-to-drag ratio is high. So, a positive $\beta_{mean}$ should be selected. The inner wing flapping angle $\beta_{in\_max}$ can adjust the overall unsteadiness of the wing and the thrust. The initial geometric twist angle can be matched with the average folding angle $\beta_{mean}$ to maintain the airfoil’s effective angle of attack within a reasonable range during upward and downward flapping.

## Figures and Tables

**Table 1:** Combinations of different kinematic parameters.

	$\beta_{in\_max}=20{}^{\circ}$ $\beta_{mean}=0{}^{\circ}$	$\beta_{in\_max}=20{}^{\circ}$ $\beta_{mean}=30{}^{\circ}$	$\beta_{in\_max}=30{}^{\circ}$ $\beta_{mean}=0{}^{\circ}$	$\beta_{in\_max}=30{}^{\circ}$ $\beta_{mean}=30{}^{\circ}$
$\phi_{\beta }=90^{{}^{\circ}}$	Case 1	Case 2	Case 3	Case 4
$\phi_{\beta }=45^{{}^{\circ}}$	Case 5	Case 6	Case 7	Case 8
$\phi_{\beta }=0^{{}^{\circ}}$	Case 9	Case 10		

**Table 2:** Comparison of performance parameters.

$\theta_{geo}$	Case 1 −4	Case 2 −4	Case3 −4	Case 4 −4	Case 5 −4	Case 6 −4	Case 7 −4	Case 8 −4	Case 9 −4	Case 10 −4	Case 4 −7
$C_{l}$	0.540	0.586	0.536	0.623	0.540	0.575	0.536	0.618	0.543	0.541	0.575
$C_{t}$	0.0278	0.0274	0.0715	0.0694	0.0551	0.0520	0.112	0.104	0.0695	0.0625	0.0691
$\eta_{P}$	0.431	0.435	0.581	0.574	0.546	0.543	0.632	0.622	0.570	0.568	0.584

## Data Availability

The data can be obtained upon request from the corresponding author.

## Nomenclature

$C_{l}$	lift coefficient of the wing
$C_{t}$	thrust coefficient of the wing
$C_{p}$	pressure coefficient
$\eta_{P}$	propulsion efficiency of the wing
$\beta_{in}$	flapping angle of inner wing
$\beta_{out}$	flapping angle of outer wing
$\beta_{in\_max}$	flapping amplitude of the inner wing
$\beta_{out\_max}$	flapping amplitude of the outer wing
$\beta_{mean}$	mean folding angle
$U_{tip}$	flapping velocity of the wing tip
*GJ*	torsional stiffnesses of the beam
*EI*	bending stiffnesses of the beam
$\phi_{\beta }$	phase difference between the flapping angles of the inner and outer wings
$\phi_{max}$	phase angle deviations relative to the inner wing flapping angle at maximum lift coefficient point
$\phi_{min}$	phase angle deviations relative to the inner wing flapping angle at maximum lift coefficient point
$\theta_{geo}$	initial geometric twist angle of the wing
$\theta_{eff}$	effective angle of attack of wing
$\theta_{max}$	maximum passive twisting angle of the wing
$\theta_{tip}$	passive twisting angle of the wing tip
$H_{max}$	flapping elastic amplitude of the wing
$\Phi_{\theta }$	phase angles between twisting angle and flapping motion
$\Phi_{H}$	phase angles between bending response and flapping motion
$\Phi_{up}$	velocity potential of the upper surface of the trailing edge
$\Phi_{down}$	velocity potential of the upper surface of the trailing edge
*k*	reduced frequency
St	Strouhal number
